# Optical Fiber-Based Steady State and Fluorescence Lifetime Spectroscopy for Rapid Identification and Classification of Bacterial Pathogens Directly from Colonies on Agar Plates

**DOI:** 10.1155/2014/430412

**Published:** 2014-09-29

**Authors:** Fathi Awad, Chandrasekaran Ramprasath, Narayanasamy Mathivanan, Prakasa Rao Aruna, Singaravelu Ganesan

**Affiliations:** ^1^Department of Medical Physics, Anna University, Chennai 600025, India; ^2^Department of Medical Physics, Red Sea University, P.O. Box 24, Port Sudan, Sudan; ^3^Centre for Advanced Studies in Botany, University of Madras, Chennai 600025, India

## Abstract

Fluorescence spectroscopy was examined as a potential technique for identification and classification of bacterial pathogens. Colonies of *Staphylococcus aureus, Pseudomonas aeruginosa, Salmonella typhi,* and *Klebsiella pneumoniae* on agar plates were measured directly using a laboratory spectrofluorimeter coupled with optical fiber. Steady state fluorescence spectra were collected following excitation at 280 nm (tryptophan) and 380 nm (NADH). Results showed that fluorescence lifetime decays of tryptophan at 280 nm excitation from the four organisms were best described with triexponential fit and it reveals the existence of different protein conformation. The emission spectroscopy of the four bacteria at 380 nm excitation (NADH) provided better classification (100% of original grouped cases correctly classified and 98.1% of cross-validated grouped cases correctly classified) than that of 280 nm excitation (tryptophan). Our results demonstrated that optical fiber-based fluorescence identification and classification of bacteria is rapid, easy to perform, and of low cost compared to standard methods.

## 1. Introduction

Fast and exact identification of microorganisms in general has been shown to have a major impact in various fields of research and industry; for example, rapid and reliable methods are needed for characterization of relevant microorganisms in medical laboratories, pharmaceutical production, and food processing technology [1, 2]. Traditional methods for bacterial identification based on morphology and biochemical tests such as bacterial cultures, DNA-based methods, and antibody-based detection scheme are laborious and reagents- and time-intensive [3]. Fast methods such as flow cytometry, direct epifluorescent filter technique (DEFT), ATP-based bioluminescence, matrix-assisted laser desorption ionization time of flight mass spectrometry (MALDI-TOF MS) [4, 5], and impedancemetry were developed for identification and classification of bacteria [6]. However, these methods are also reagent- and time-intensive. Molecular methods based on polymerase chain reaction (PCR) and others techniques are fast and much more accurate, but they are expensive to perform [7–9]. Raman and Fourier transform infrared (FT-IR) spectroscopy has been presented as a substitute to the above mentioned methods [10–13], but although reliable, it needs expensive dedicated equipment and trained personnel [14].

Fluorescence based techniques have been shown to provide the most sensitive detection of biomolecules. The in situ measurement capability with no sample contact, short collection time, and the capability of scanning large areas/volumes continuously renders fluorescence methods attractive properties for the detection [15], identification [16, 17], and classification [18] of microorganisms. Intrinsic fluorescence spectroscopy has been shown to allow the detection and differentiation of viable but not culturable (VBNC) bacteria, thus overcoming the limitations of most actually used diagnostic techniques for VBNC gram negative bacteria [19]. Furthermore, there are increasing interests in the use of fluorescence lifetime in basic and applied sciences, as it is independent of fluorophore concentration and perturbation conditions, such as duration of light exposure or the excitation wavelength.

Living bacteria retain a variety of intracellular biomolecules, such as tryptophan, NADH, FAD, other amino acids (tyrosine and phenylalanine), and nucleic acids that emit photons following excitation at specific wavelengths in the ultraviolet region [19]. Tryptophan fluorescence is the most intense and shows broad emission from 320 to 400 nm. Since various types of bacteria contain different amounts of fluorophores in different microenvironments, steady state, and fluorescence lifetime spectroscopy may be considered for discrimination between different types of bacteria [2, 20].

Various earlier studies have proposed the use of fluorescence spectroscopy for microbial identification and classification. Sohn et al. [18] reported the successful use of steady state fluorescence coupled with principal component analysis (PCA) for detection and differentiation of* Escherichia coli*,* Salmonella*, and* Campylobacter* using synchronous scan and excitation at 225 nm and 280 nm. Giana et al. [21] studied the identification of* Escherichia coli*,* Enterococcus faecalis*, and* Staphylococcus aureus*, using excitation at 410 nm and 430 nm. The intrinsic steady state and fluorescence lifetime characteristics of tryptophan for* staphylococcus epidermidis*,* pseudomonas fluorescence*,* Enterobacter cloacae*,* Escherichia coli*, and* Bacillus subtilis* were investigated by Dalterio et al. [20]. The authors also reported that the rather small range of difference in the fast lifetimes (1.93–2.27 ns) and slow lifetimes (5.08–6.02 ns) may limit the use of these parameters alone for the identification of bacteria. For all these studies, fluorescence spectra were acquired for bacteria grown on a liquid medium and suspended in saline solution after centrifugation and washing. Recently, Belal et al. [6] reported the use of optical fiber-based synchronous fluorescence spectroscopy to discriminate colonies of* Pseudomonas* and related reference strains directly on agar plate. The authors also used classic fluorescence at 250 nm and 340 nm excitation for aromatic amino acids and nucleic acids (AAA + NA) and nicotinamide adenine dinucleotide (NADH), respectively. However, the literature contains very few reports on the use of optical fiber-based fluorescence spectroscopy for bacterial identification and classification. To the best of our knowledge, there is no published study that investigated the use of optical fiber-based fluorescence lifetime spectroscopy of bacteria directly from colonies on agar plates.

In this context, the aim of the present study was to investigate the use of optical fiber-based fluorescence spectroscopy for rapid identification and classification of* Staphylococcus aureus* (*S. aureus*),* Pseudomonas aeruginosa* (*P. aeruginosa*),* Salmonella typhi* (*S. typhi*), and* Klebsiella pneumoniae* (*K*.* pneumoniae*) directly from colonies on agar plates. Steady state spectra were obtained following excitation at 280 nm (tryptophan) and 380 nm (NADH). The obtained spectra were processed using principal component analysis (PCA) to classify the four bacteria according to their genus. Fluorescence lifetime decays of tryptophan (at 280 nm excitation) from bacterial colonies were used to extract additional information about their microenvironment.

## 2. Materials and Methods

### 2.1. Preparation of Bacterial Samples

*S. aureus* (ATCC 6538),* P. aeruginosa* (ATCC 10145),* S. typhi* (ATCC 12600), and* K. pneumoniae* (ATCC 13883) were obtained from the Centre for Advanced Studies in Botany, University of Madras. The bacteria were maintained in tryptic casein agar (Himedia, Mumbai, India) in stock culture plates until it is used for the experiments. Before the experiments, bacteria were grown in Muller-Hinton broth (MHB) (Himedia, Mumbai, India) at 37°C for 16 h with shaking at 200 rpm in LS 500 incubator/shaker (Neolab, Mumbai, India). The bacterial pellet was harvested by centrifugation at 10,000 rpm for 10 min. Subsequently, the bacterial cells washed using normal saline (NaCl 9 g/L) and grown on agar plates with nutrient agar medium (Himedia, Mumbai, India) for 24 h. For testing the reproducibility, each bacterium culture was grown on subsequent days (for 3 days) in agar plate. For each plate, 4 colonies were measured, yielding a total of 12 samples for each bacterium.

### 2.2. Steady State Fluorescence Measurements

Steady state fluorescence spectra of the bacterial colonies were recorded using Fluorolog spectrofluorometer (Flurolog, HORIBA instruments Inc., USA) coupled with optical fiber. The optical fiber was placed at 9 mm above the colonies [6]. The excitation source (450 W Xenon lamp) is coupled to the double excitation monochromator to obtain the light of a desired wavelength. The fluorescence emission was collected using the double emission monochromator connected to a fast response red sensitive PMT (Hamamatsu Photonics, Japan). The excitation and emission monochromators gratings have a groove density of 1200 grooves/mm. The collected signal is transferred to the PC using an RS 232 interface. The data were processed using FluorEssence software powered by Origin 7.5. The fluorescence emission spectra of tryptophan (300–540 nm, resolution: 1 nm, slits: 5 nm) and NADH (400–740 nm, resolution: 1 nm, slits: 5 nm) were recorded with excitation set at 280 nm and 380 nm, respectively.

### 2.3. Fluorescence Lifetime Measurements

Fluorescence lifetime measurements of tryptophan were obtained using Time Correlated Single Photon Counting System (TCSPC, HORIBA instruments Inc., USA) coupled with optical fiber. The optical fiber was placed at 9 mm above the colonies [6]. The bacterial colonies were excited using 280 nm Nano LED (Pulse width: <1 ns), with a fast response red sensitive PMT (Hamamatsu Photonics, Japan) detector. The maximum emission wavelength for each bacterium was set as it was observed in the steady state measurements. The electrical signal was amplified by a TB-02 pulse amplifier (Horiba) and fed to the constant fraction discriminator (CFD, Phillips, The Netherlands). The first detected photon was used as a start signal by a time-to-amplitude converter (TAC), and the excitation pulse triggered the stop signal. The multichannel analyzer (MCA) recorded repetitive start-stop signals from the TAC and generated a histogram of photons as a function of time-calibrated channels until the peak signal reached 5,000 counts. Lifetime components were extracted using decay analysis software (DAS6 V6.0, Horiba). The goodness of fit was judged by chi-square values and Durbin-Watson parameters, as well as visual observations of residuals, autocorrelation functions, and fitted line.

### 2.4. Data Processing and Principal Component Analysis

Basically in PCA the maximum variance of the data is concentrated in a decreased number of independent variables termed as principal components (PCs) [21]. The most pronounced variation in the spectral data arises from the differences in the nature of the samples, which means a few variables. Normally, the first PCs contain significant part of the variance (greater differentiation among spectra). However, the last PCs contain only uncorrelated information or noise.

Steady state emission spectra, as acquired with spectrometer, were imported to SPSS (SPSS Incorporated, Chicago, Illinois) and analyzed by principal component analysis (PCA). PCA was applied separately for each fluorophore (tryptophan and NADH). The data were introduced into predefined groups (one group was one genus) and four groups were created (*S. aureus*,* P. aeruginosa*,* S. typhi*, and* K. pneumoniae*). Thus, the data matrix for each fluorophore consisted of 48 observations (4 strains × 12 spectra per strain). In order to obtain the best spectral differentiation among the four bacteria, it used the first three PCs that contain about 90% of the variance of the spectra.

## 3. Results and Discussion

### 3.1. Steady State Fluorescence Measurements

Current traditional diagnostic methods and techniques of microorganisms such as bacteria need normally at least one day. Antibiotic sensitivity test should also be performed to select the effective antibiotic for treating infection and it takes one more day. These tests cause delay in start of the required treatment. Normally, the physicians prescribe broad spectrum antibiotics that are unneeded and expensive for the patients. Moreover, microorganism may develop resistance for this antibiotic treatment. Hence, fast diagnosis of microorganism and starting the specific treatment are recommended.

Although, fluorescence-based detection and characterisation have been investigated for environmental or food-contaminant microbes, no apparatus has yet been developed for bacterial detection for clinical application at the macroscale using standoff, “remote sensing,” detection [22].

It was reported that many bacteria exhibited fluorescence from proteins and flavin [20]. At excitation wavelengths near 295 nm, the amino acid tryptophan only is excited and at excitation lesser than 295 nm the fluorescence from tryptophan and tyrosine is possible. However, the energy transfer from tyrosine to tryptophan may take place simultaneously [23].

**Figure 1:**
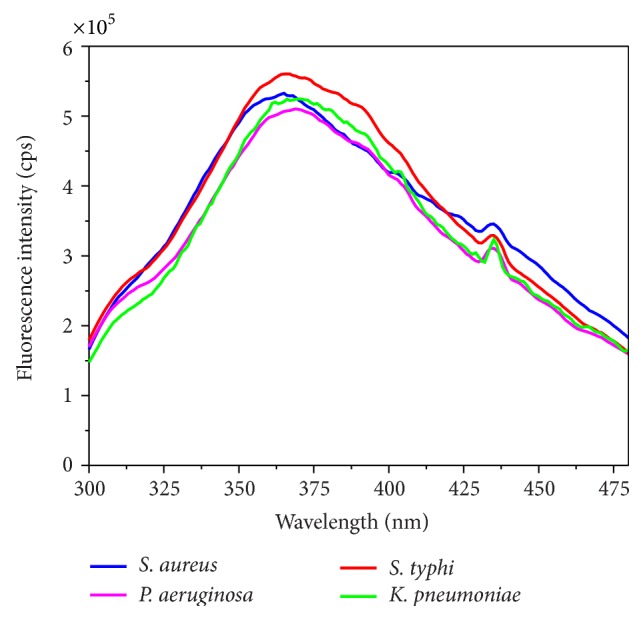
Tryptophan average steady state emission spectra (*λ*_exc_ = 280 nm) for* S. aureus*,* P. aeruginosa*,* S. typhi*, and* K. pneumonia.*

Figure 1 shows steady state emission spectra at 280 nm excitation for* S. aureus*,* P. aeruginosa*,* S. typhi*, and* K. pneumoniae*. As the proposed method is fast, the measurements were carried out directly in agar plate to minimize the time for sample preparation. The emission maxima of tryptophan from* S. aureus*,* P. aeruginosa*,* S. typhi*, and* K. pneumoniae* are shown in Table 1. The exact location of the maximum emission depended on the organisms as previously reported by Leblanc and Dufour [16] and Ammor et al. [24].

Good reproducibility was obtained for tryptophan fluorescence measurements for the same strain of bacteria that were grown on different days.

**Figure 2:**
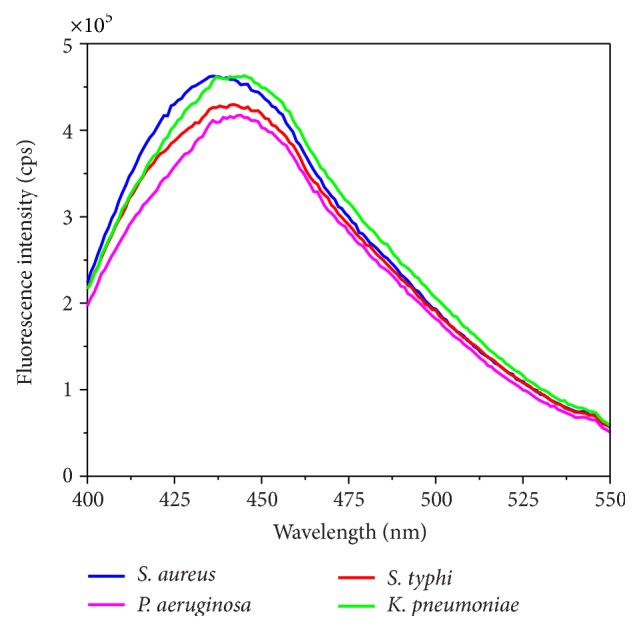
NADH average steady state emission spectra (*λ*_exc_ = 380 nm) for* S. aureus*,* P. aeruginosa*,* S. typhi*, and* K. pneumonia.*

In addition to the emission characteristics of key amino acids, the native fluorescence from the enzyme NADH is also considered for the identification and classification of bacterial strains. NADH is a metabolic coenzyme (the reduced form for a pyridine nucleotide) which is electron carriers assuming a continual input of free energy through cellular metabolism [24]. The average emission spectra of NADH from the four organisms exhibited a considerable variation as shown in Figure 2 which may be attributed to the variation of this metabolic coenzyme within the measured colonies. The peak maxima of NADH are shifted to either higher or lower wavelengths depending on the bacteria as shown in Table 1. Good reproducibility was obtained for NADH steady state fluorescence measurements for the same strain of bacteria that were grown on different days. From Table 1 it can be observed that the emission maxima from* S. aureus* exhibited greater spectral shift when compared to* P. aeruginosa*,* S. typhi*, and* K. pneumoniae*. This shift may be attributed to difference in the protein structure between gram positive and gram negative bacteria.

### 3.2. Fluorescence Lifetime Measurements

Time-resolved fluorescence anisotropy studies on tryptophan containing protein have demonstrated that in some proteins the indole group exhibits a considerable freedom of rotation occurring in a timescale of subnanosecond [25]. These rotations place the indole group in different microenvironments and add a distribution of interactions with nearest-neighbor amino acid side chains [26]. The differing microenvironments provide a range of quenchers for the tryptophan residues which cause a distribution of fluorescence decay times and hence, a multiexponential decay kinetics [25]. Conventionally, each decay is analyzed in terms of exponential components and the values of the preexponential factors and decay rates of each component are related to a particular conformation and to the relative population of each conformation [27].

Optical fiber-based fluorescence lifetime measurements are being researched by several groups [28–30]. Moreover, measurement of fluorescence decay lifetime, as opposed to steady state, has special advantages because it is relatively independent of light scattering, signal amplitude fluctuations, and fluorophore concentration [28]. Further, advantages such as its high sensitivity to local microenvironment (temperature, pH, viscosity, and polarity) and capability to study fluorophores exhibiting the same emission wavelength with different fluorescence decay make this technique more attractive [29]. In this study, fluorescence lifetime decays of tryptophan from different bacterial colonies were used to extract additional information about their microenvironment.

**Figure 3:**
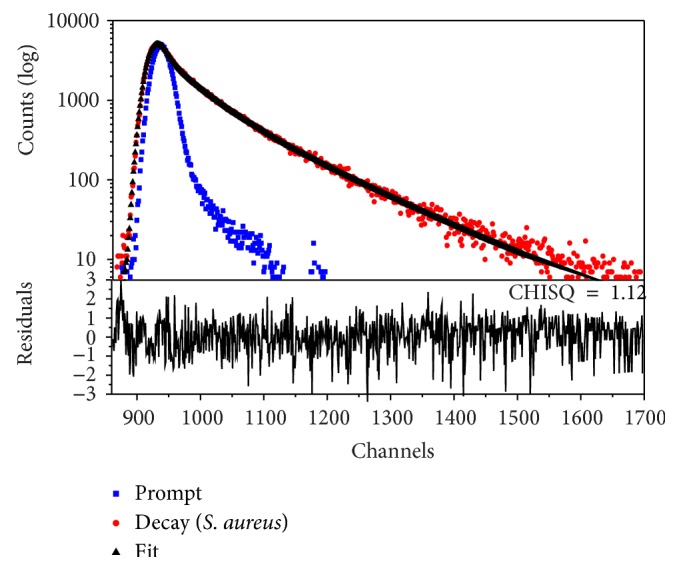
Typical tryptophan lifetime decay from* S. aureus* at 280 nm excitation.

Table 2 lists tryptophan average fluorescence lifetime components and corresponding amplitudes from* S. aureus*,* P. aeruginosa*,* S. typhi*, and* K. pneumoniae* obtained with excitation at 280 nm. Typical decay curves for tryptophan fluorescence from the four bacteria are shown in Figure 3. All lifetime decays were best fitted with triexponential fit. Dalterio et al. [20] studied the fluorescence lifetime characteristics of tryptophan for* Staphylococcus epidermidis*,* Pseudomonas fluorescence*,* Enterobacter cloacae*,* Escherichia coli*, and* Bacillus subtilis* suspended in saline solution. The authors also reported that the rather small range of difference in the fast lifetime components (1.93–2.27 ns) and slow lifetime components (5.08–6.02 ns) may limit the use of these parameters alone for identification of bacteria.

From Table 2 it is observed that the fast lifetime component (*τ*_1_) varied from 0.18 ns for* P. aeruginosa* to 0.29 ns for* K. pneumoniae.* The intermediate lifetime component varied from 1.60 ns for* P. aeruginosa* to 2.17 ns for* K. pneumoniae*. The slow lifetime component exhibited a considerable variation (4.16 ns for* S. aureus* to 5.65 ns for* K. pneumoniae*), for example, the measured slow lifetime from the four different bacterial strains in the order of* S. aureus* <* P. aeruginosa* <* S. typhi* <* K. pneumoniae*. The observed differences in fast, intermediate, and slow lifetime component could be due to the presence of different microenvironment such as viscosity and chromophores of charged group accomplished by tryptophan residues in proteins [20, 31]. It was reported that single tryptophan and its residues conformers itself were strongly influenced by their microenvironment; hence conformational changes may result in changes over lifetime [32].

### 3.3. Principal Component Analysis

To assess the classification potential of the steady state fluorescence spectra at genus level, PCA was performed separately on tryptophan and NADH spectra. The classification results of the four bacteria for tryptophan and NADH are shown in Table 3. At 280 nm excitation, 96.2% of correct classification for original and cross-validated grouped cases was observed. In addition, it is noted that there is an overlap of two cases (one* P. aeruginosa* strain classified as* S. typhi* and one* S. typhi* strain classified as* P. aeruginosa*). On the other hand, NADH showed the highest percentage of good classification with 100% of original grouped cases correctly classified and 98.1% of cross-validated grouped cases correctly classified. The PCA results clearly indicate that fluorescence spectroscopy of NADH may be considered as a tool to classify the bacteria under in situ condition. This may be due to changes in the metabolic activity between the strains.

**Figure 4:**
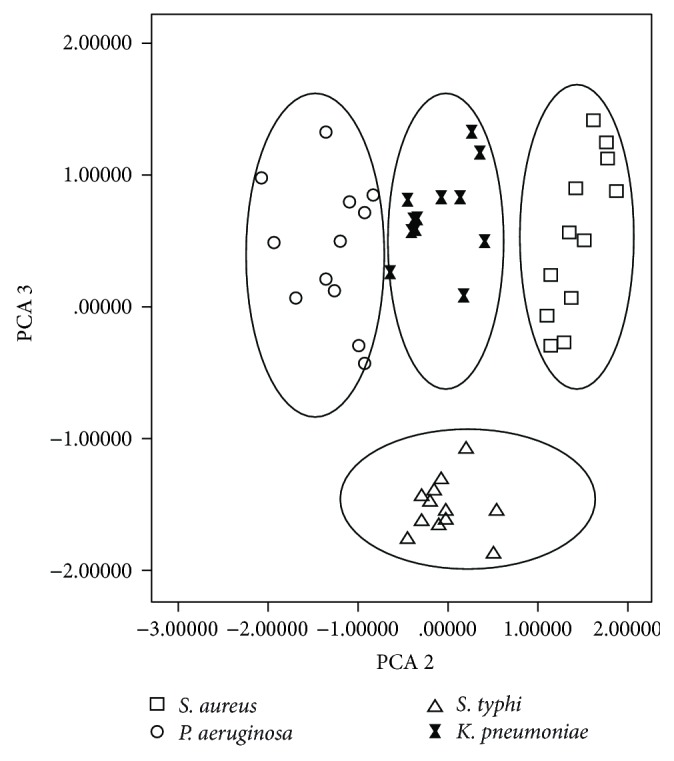
Principal component analysis score plots for* S. aureus*,* P. aeruginosa*,* S. typhi*, and* K. pneumoniae*. *λ*_exc_ = 380 nm (NADH).

The PCA score plots for steady state emission spectra of the four bacteria from NADH are shown in Figure 4. Four groups equivalent to the four bacteria were noticeably separated from each other suggesting that fluorescence spectra collected directly from bacterial colonies can be used as fingerprints as had been previously reported for bacteria suspended in a liquid medium.

Bacterial colonies are extremely heterogeneous in terms of genotypic and phenotypic status and physiologic stat [33]. Furthermore, the complex matrices that enclose bacterial cells contain numerous metabolites produced during bacterial growth. The heterogeneity within colonies could be expected to interrupt the reproducibility of the investigated technique. However, the results clearly demonstrate the reproducibility and reliability of the method.

## 4. Conclusion

The use of optical fiber steady state fluorescence spectroscopy for bacterial identification and classification is fast and simple. The results can be obtained without addition of reagents or manual preparation of cells, eliminating all human errors or the contamination during preparation of the bacterial samples.

Fluorescence lifetime decays of tryptophan from* S. aureus*,* P. aeruginosa*,* S. typhi*, and* K. pneumoniae* colonies were best described with triexponential fit and it reveals the existence of different protein conformation. The emission spectroscopy of the four bacteria at 380 nm excitation provided better classification than that at 280 nm excitation.

As this method is simple, sensitive, and of low cost, further studies on large scale should be performed to validate fluorescence spectroscopy as identification tool for microorganisms.

## Figures and Tables

**Table 1:** Emission maxima of tryptophan and NADH from *S. aureus, P.aeruginosa, S. typhi,* and *K. pneumonia*.

Sample	Tryptophan (*λ*_exc_ = 280 nm)	NADH (*λ*_exc_ = 380 nm)
*S. aureus *	363	436
*P. aeruginosa *	368	444
*S. typhi *	366	442
*K. pneumoniae *	368	445

**Table 2:** The fast (*τ*_1_), intermediate (*τ*_2_), and slow (*τ*_3_) lifetime components and their relative amplitudes from *S. aureus, P. aeruginosa, S. typhi,* and* K. Pneumoniae* at 280 nm excitation.

Sample	Fast component		Intermediate component		Slow component		CHISQ
	*τ*_1_ (ns)	*A*_1_ (%)	*τ*_2_ (ns)	*A*_2_ (%)	*τ*_3_ (ns)	*A*_3_ (%)	
*S. aureus *	0.19 ± 0.02	0.24 ± 0.02	1.68 ± 0.07	0.26 ± 0.02	4.16 ± 0.09	0.50 ± 0.01	1.08 ± 0.05
*P. aeruginosa *	0.18 ± 0.03	0.27 ± 0.01	1.60 ± 0.10	0.25 ± 0.02	4.57 ± 0.07	0.48 ± 0.02	1.05 ± 0.06
*S. typhi *	0.21 ± 0.03	0.28 ± 0.06	1.86 ± 0.08	0.33 ± 0.06	5.02 ± 0.10	0.39 ± 0.02	1.06 ± 0.05
*K. pneumoniae *	0.29 ± 0.04	0.24 ± 0.01	2.17 ± 0.12	0.34 ± 0.02	5.65 ± 0.05	0.42 ± 0.02	1.05 ± 0.06

(ns): nanosecond.

**Table 3:** Percentage of correct classification according to the confusion matrix resulting from PCA analysis performed on tryptophan and NADH emission spectra collected from *S. aureus*, *P. aeruginosa, S. typhi,* and *K. pneumonia*.

Fluorophore	Index of good classification according to the confusion matrix resulting from PCA	
	Original (%)	Cross-validated
Tryptophan	96.2	96.2
NADH	100	98.1
